# p53 Configures the G2/M arrest response of nucleostemin-deficient cells

**DOI:** 10.1038/cddiscovery.2015.60

**Published:** 2015-11-23

**Authors:** G Huang, L Meng, RYL Tsai

**Affiliations:** 1 Institute of Biosciences and Technology, Texas A&M University Health Science Center, Houston, TX, USA; 2 Department of General Surgery, The Fifth Affiliated Hospital of Guangzhou Medical University, Guangzhou 510700, China; 3 Department of Molecular and Cellular Medicine, Texas A&M University Health Science Center, College Station, TX, USA

## Abstract

Nucleostemin (NS) protects the genome from replication-induced DNA damage and has an indispensable role in maintaining the continuous proliferation of both p53-wild-type and mutant cells. Yet, some outcomes of NS-deficient cells appear to be shaped by their p53 status, which stimulates conflicting claims on the role of p53 in executing the NS function. This disparity was conveniently attributed to the usual suspect of cell-type variations. To provide a definitive resolution, we investigated the interplay between NS and p53 in two pairs of isogenic cells, that is, genetically modified mouse embryonic fibroblast (MEF) cells and HCT116 human colon cancer cells. In MEF cells, p53 deletion further compromises rather than rescues the proliferative potential of NS-depleted cells without changing their G2/M arrest fate before prophase entry. The detrimental effect of p53 loss in NS-depleted MEF cells correlates with a dramatic increase of polyploid giant cells (PGCs) (up to 24%), which indicates aberrant mitosis. To determine how p53 shapes the response of cells to NS depletion at the molecular level, we showed that p53 turns on the expression of reprimo and MDM2 in NS-deficient MEF cells. In absence of p53, NS-deficient MEF cells exhibit increased levels of phosphorylated cdc2 (Y15) protein and cyclin B1. In cancer (HCT116) cells, NS loss leads to G2/M arrest under both p53wt and p53ko conditions and increases phosphorylated cdc2 more in p53ko than in p53wt cells, as it does in MEF cells. Unlike its effect in MEF cells, NS depletion decreases tumor growth and increases the expression of reprimo and cyclin B1 in a p53-independent manner in HCT116 cells. Our data indicate that the p53 status of NS-deficient cells orchestrates how they respond to G2/M arrest in a normal *versus* cancer cell distinct fashion.

## Introduction

Mammalian nucleostemin (NS) was first discovered as a gene more abundantly expressed by embryonic neural stem cells than their progeny^1^ and later found to be highly enriched in many stem cell types and cancer cells.^1–6^ The importance of NS has been unequivocally shown in several biological events of fundamental significance, including blastocyst formation,^7,8^ embryogenesis,^9^ postnatal tissue regeneration,^10,11^ cancer development,^5,6,12^ and reprogramming to pluripotency.^13^ The majority of NS protein is stored in the nucleolus but takes action outside the nucleolus via a GTP-controlled shuttling mechanism.^14,15^

Earlier studies indicated that NS (mostly that of the mammal) and its paralogue is physically associated with MDM2 and functionally linked to p53 inactivation.^1,16–19^ If the MDM2-p53 regulation represents a major target of NS action, one may logically infer that p53 loss should partially or completely reverse the detrimental outcome of NS deletion. Yet, several studies have demonstrated that NS remains indispensable for the survival and continuous proliferation of p53-null normal or cancer cells.^8,9,20^ We recently discovered a key role of NS in reducing the amount of DNA damage accumulated during the S phase.^4,9,11,21^ In accord with the p53 independency of NS, deletion of NS leads to DNA damage to the same extent in p53-wild-type (p53wt) MEF cells as in p53-null (p53ko) cells.^9^ Based on the evidence reported so far,^22^ we believe that the essential function of NS is best captured by its genome-protective activity, whereas its MDM2 regulatory function occurs mainly in the context of mitosis or nucleolar stress, when the NS protein is released *en masse* from the nucleolus to the nucleoplasm.

This model, however, does not preclude the possibility that p53 may still be involved in guiding the ensuing events after NS depletion. Indeed, we found that the growth of NS-deficient MEF cells becomes more severely prohibited without p53 than with p53.^9^ This paradoxical finding certainly refutes the idea that the obligatory function of NS depends on p53 inhibition, but more importantly it supports that the p53 status may influence how cells respond to NS depletion. To date, it is not entirely clear how exactly the outcome of NS-deficient cells is shaped by their p53 status, considering the many genetic variations that exist between the difference cell models used in different studies. To overcome this challenge, we used two pairs of isogenic cell models to understand the interplay between NS and p53 perturbation. Our findings provide new insight into how p53 may shape the response of NS-deficient cells to G2/M arrest.

## Results

### Loss of p53 aggravates the already diminished proliferation of NS-depleted MEF cells

**Figure 1:**
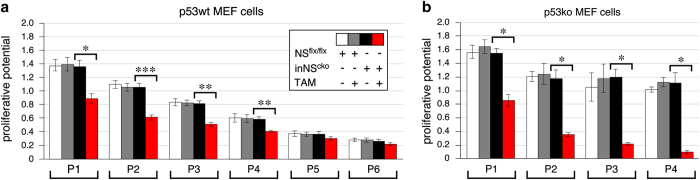
Loss of p53 decreases the proliferative potential of NS-depleted mouse embryonic fibroblast (MEF) cells. MEF cells were prepared from NS^flx/flx^ or inNS^cko^ embryos in the p53-wild-type (p53wt) (**a**) or p53-null (p53ko) (**b**) background and treated with 0.1 *μ*M tamoxifen (TAM) or DMSO from passage 1 (P1) to P4–P6. The proliferative potential was determined as the average daily cell expansion ratio during each passage. Bars and error bars represent the average and s.e.m. *P⩽0.01, **P⩽0.001 and ***P⩽0.0001.

To determine how p53 regulates the NS-knockdown (NSkd) response of normal cells, we created a tamoxifen (TAM)-inducible NS^cko^ mouse model (inNS^cko^) by introducing the Cre^ER^ transgene ^23^ into NS^flx/flx^ mice^9^ and then bred inNS^cko^ and NS^flx/flx^ mice into the p53ko background.^24^ MEF cells were harvested from E13.5 mouse embryos of four genetic backgrounds, i.e. NS^flx/flx^-p53wt, inNS^cko^-p53wt, NS^flx/flx^-p53ko, and inNS^cko^-p53ko, and cultured on a 3- day-passage schedule with or without 0.1 *μ*M TAM. Western blots confirmed a specific decrease of NS protein in TAM-treated inNS^cko^ cells compared with DMSO-treated inNS^cko^ cells or TAM-treated NS^flx/flx^ cells (Supplementary Figure S1a). The proliferative potentials of NS- and/or p53-perturbed MEF cells were determined by calculating the average daily cell expansion ratio during the 3-day period of each passage. TAM-induced NS loss decreases the proliferative potential of p53wt MEF cells by 30–40% in early passages (P1–P4) (Figure 1a). This growth-inhibitory effect of NS loss becomes indiscernible when cells enter late passages (P5–P6). Interestingly, loss of p53 increases the proliferative potential of control MEF cells (NS^flx/flx^ with/without TAM and inNS^cko^ without TAM) but reduces the proliferative potential of NS-depleted MEF cells (inNS^cko^ with TAM) (Figure 1b). This paradoxical effect of p53 deletion on control *versus* NS-deficient MEF cells becomes more evident as the passage number increases from P1 to P4 (Supplementary Figure S1b). These findings indicate that p53wt MEF cells have a higher dependency on NS at early passages than late passages, whereas p53-null MEF cells are progressively more sensitive to the anti-proliferative effect of NSkd at later passages.

### NS-deficient MEF cells arrest in the G2/M phase regardless of their p53 status

**Figure 2:**
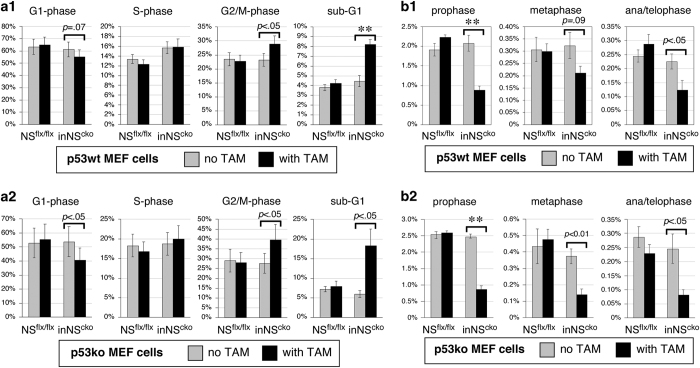
NS depletion triggers G2/M arrest in both p53wt and p53ko MEF cells.(**a**) MEF cells derived from NS^flx/flx^ or inNS^cko^ embryos in the p53wt or p53ko background were treated with DMSO (gray bars) or TAM (0.1 *μ*M, black bars) for 4 days at P2 and analyzed by propidium iodide (PI)-labeled flow cytometry. The averages for p53wt cells (*n*=9) and p53ko cells (*n*=4) were shown in (**a1**) and (**a2**), respectively. (**b**) The percentages of prophase, metaphase, anaphase, and telophase cells were quantified by anti-pH3 staining in p53wt (**b1**) and p53ko (**b2**) MEF cells from eight experiments. See Figure 1 for description regarding error bars and asterisks.

To determine whether the cell cycle outcome of NS-depleted MEF cells depends on p53, we treated P2 MEF cells with or without TAM (0.1 *μ*M) for 4 days and analyzed their cell cycle profiles. Propidium iodide (PI)-labeled studies showed that TAM-induced NS depletion in p53wt MEF cells (inNS^cko^+TAM) causes a slight decrease in G1 cells and an increase in G2/M and sub-G1 (3–4%) cells (Figure 2a1). This profile indicates a G2/M arrest and is consistent with a partial NSkd effect seen in MDA-MB-231 breast cancer cells.^25^ In p53ko MEF cells, NS depletion also results in a G2/M arrest phenotype and a sub-G1 cell increase (~12%) (Figure 2a2). To determine whether NS-depleted cells were checked in the G2 or M phase, we quantified the percentages of MEF cells in the mitotic prophase, metaphase, anaphase, and telophase by their expression of phosphorylated histone H3 (pH3) at serine 10, which occurs exclusively during mitosis in mammalian cells.^26,27^ In p53wt MEF cells, decreases in the prophase, metaphase, anaphase, and telophase cells were specifically noted in the TAM-treated inNS^cko^ cells compared with the controls (Figure 2b1). In p53ko MEF cells, the same defect in prophase entry occurs following NS depletion (Figure 2b2). These results indicate that a partial loss of NS results in G2 arrest before mitotic entry and that this event occurs regardless of the p53 status.

### Loss of p53 leads to a dramatic increase of PGCs in NS-depleted MEF cells

**Figure 3:**
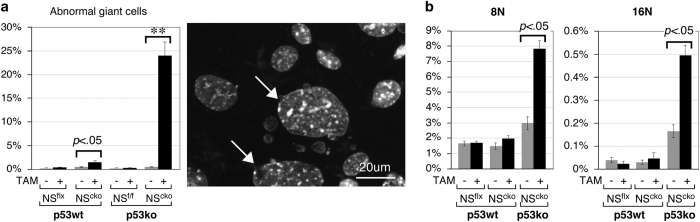
Deletion of p53 enhances polyploid giant cell formation in NS-depleted MEF cells. (**a**) DAPI staining reveals a dramatic increase in cells with an abnormal giant nuclear morphology (nuclear diameter ⩾40 *μ*m) in NS-p53 double-knockout MEF cells (P3). (**b**) The amounts of polyploid cells (8 N and 16 N) in P2 MEF cells were measured by PI-labeled flow cytometry. NS^flx/flx^ and inNS^cko^ are represented by NS^flx^ and NS^cko^, respectively. See Figure 1 for description regarding error bars and asterisks.

The slight difference in the degree of G2/M arrest between p53wt-NSkd and p53ko-NSkd cells does not match the major disparity of their proliferative potentials. To search for what might account for the p53ko-aggravated effect in NS-depleted cells, we determined the percentages of senescent cells in P3 MEF cells by SA-β-Gal staining and found that NS depletion does not increase SA-β-Gal^+^ cells in the presence of p53 but does so in the absence of p53. However, the increase of SA-β-Gal^+^ cells by NSkd in p53ko cells only amounts to <1% of the total cells (data not shown), which hardly matches the scale of the decrease in cell proliferation. In contrast, DAPI staining revealed that 24% of the NSkd-p53ko MEF cells show abnormal giant nuclear morphology, which is significantly more abundant than that of NSkd-p53wt cells (1.5%) or NS-wild-type cells (<0.5%) (Figure 3a). Many abnormal giant cells are associated with small satellite nuclei or lobulated nuclei. PI-labeled flow cytometry confirmed a significant increase of polyploid cells (8 N and 16 N) in NSkd-p53ko MEF cells (Figure 3b). These results indicate that p53 deletion precipitates the frequency of mitotic aberration and the formation of PGCs in NSkd MEF cells.

### NS depletion elicits distinct molecular responses in p53ko and p53wt MEF cells

**Figure 4:**
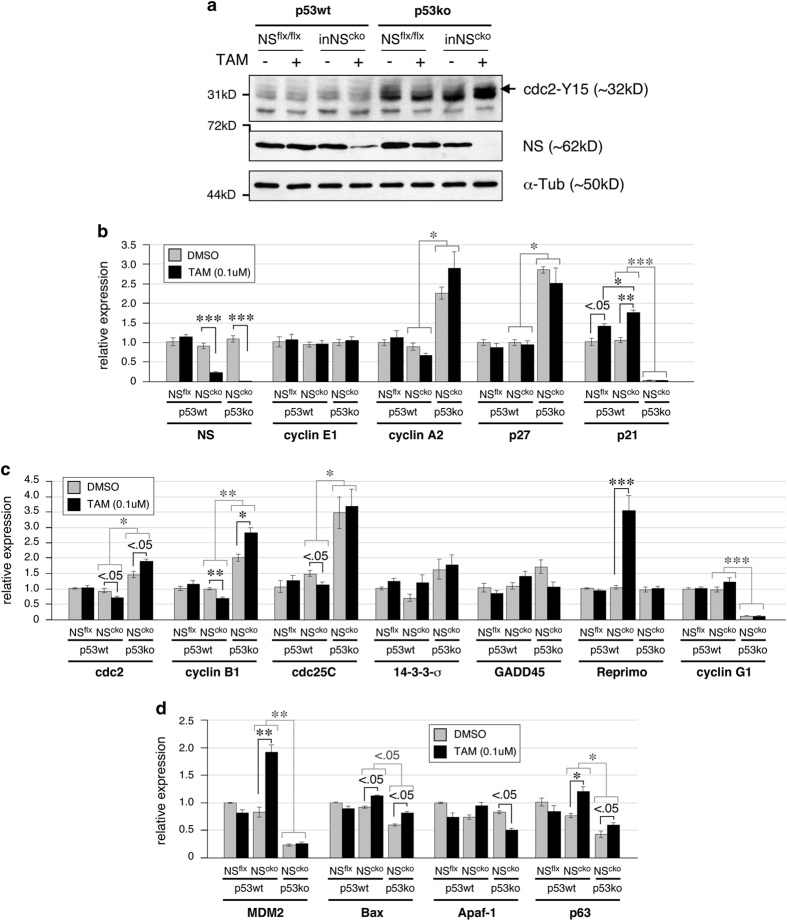
Molecular responses to NS depletion in p53wt and p53ko MEF cells. MEF cells were prepared from four genetic models. P2 cells were treated with DMSO (gray bars) or TAM (0.1 *μ*M, black bars) for 4 days and collected for western blotting (**a**) or quantitative RT-PCR analyses (**b**–**d**). The levels of gene products that are involved in G1/S control (**b**), G2/M control (**c**), apoptosis, and feedback regulation of p53 (**d**) were measured by qRT-PCR (*n*=6). See Figure 1 for description regarding error bars and asterisks.

Entry into the M phase is driven by the dephosphorylation of phosphorylated cdc2 and a surge of cyclin B1. Dephosphorylation of cdc2 is triggered by nuclear translocation of cdc25C phosphatase. To gain insight into how p53 regulates the mitotic blockage of NSkd cells, we first measured the level of phosphorylated cdc2 (Tyr15) in response to NS deletion. Our results showed that NS depletion increases the amount of phosphorylated cdc2 protein in p53ko MEF cells but not in p53wt cells (Figure 4a), suggesting that p53 may direct NS-deficient cells toward a G2/M arrest pathway different from that of p53ko cells. To identify the potential targets of p53, we used the quantitative RT-PCR assay to measure the transcript levels of several gene products involved in G1/S arrest, G2/M arrest, apoptosis, or p53 feedback control. We first confirmed that TAM treatment decreases NS transcripts by 74% in p53wt cells and by >90% in p53ko cells (Figure 4b). The amounts of several G1/S regulators, including cyclin E1, cyclin A2, and p27^kip^, show no NS-dependent changes in either p53wt or p53ko cells, whereas p21^cip^ is increased only in p53wt cells in a TAM-dependent but NS-independent manner (Figure 4b). Compared with p53wt cells, the baseline levels of cyclin A2 and p27^kip^ in p53ko cells are higher, regardless of their NS status, and their baseline level of p21^cip^ is lower (Figure 4b, gray asterisks). For genes that regulate the G2/M transition, we found that NS loss increases the transcript levels of cdc2 and cyclin B1 only in p53ko cells, but increases the level of reprimo only in p53wt cells (Figure 4c). Compared with p53wt cells, p53ko cells express a higher baseline level of cdc2, cyclin B1, and cdc25C and a lower baseline level of cyclin G1. In addition, NS deletion increases the level of MDM2 only in p53wt cells and the levels of Bax and p63 in both p53wt and p53ko cells (Figure 4d). These results identify p53-dependent *versus* independent changes following NS loss. Specifically, NS depletion upregulates the expression of reprimo and MDM2 in p53wt MEF cells, the levels of phosphorylated cdc2 and cyclin B1 in p53ko cells, and the expression of Bax and p63 in both p53wt and p53ko cells.

### Loss of NS blocks both HCT116-8 and HCT116-2 cells at G2/M and reduces their clonogenic survival

**Figure 5:**
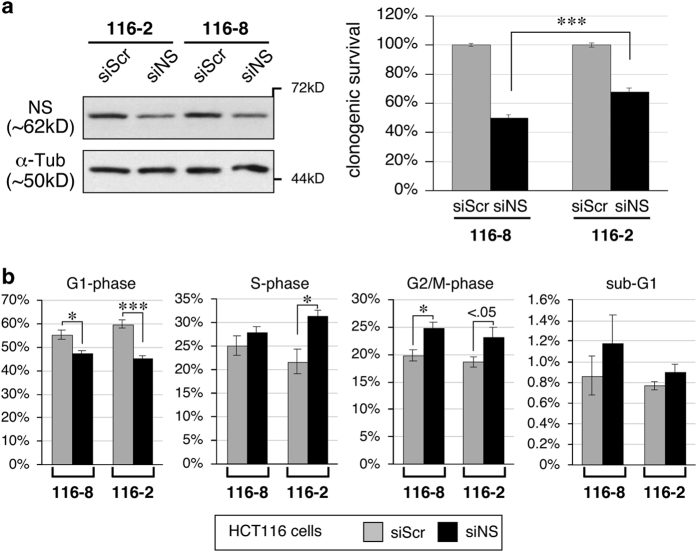
NS depletion reduces the clonogenic survival of HCT116 cells and triggers G2/M arrest in a p53-independent manner. (**a**) Western blots (left panel) and clonogenic survival (right panel) of HCT116-8 (p53wt) and HCT116-2 (p53ko) cells in response to control (siScr) or NS-specific (siNS) siRNA treatment. NS was detected by the Ab138 antibody. (**b**) Flow cytometry analysis of cell cycle profiles (*n*=9). See Figure 1 for description regarding error bars and asterisks.

p53 mutation is commonly seen in human cancer. We used two isogenic human colorectal cancer cells, HCT116-8 (p53wt) and HCT116-2 (p53ko) cells, to determine how p53 influences the clonogenic survival and cell cycle arrest phenotype of NS-depleted cancer cells. We first showed that siNS treatment reduces NS protein comparably in HCT116-8 and HCT116-2 cells (Figure 5a, left panel). NSkd by siNS decreases the survival of both p53wt and p53ko HCT116 cells (Figure 5a, right panel). Unlike MEF cells, loss of p53 does not further compromise the survival of HCT116 cancer cells, if not slightly improves it (Figure 5a). Cell cycle profile analyses showed that NS depletion decreases the G1 cell percentage and increases the G2/M cell percentage in both HCT116-8 and HCT116-2 cells to similar extents (Figure 5b). We also observed an increase in the S-phase cell percentage in HCT116-2 cells but no significant increase in the sub-G1 cell percentage in either cell type following NSkd.

### Molecular responses of p53wt and p53ko cancer cells to NS depletion

**Figure 6:**
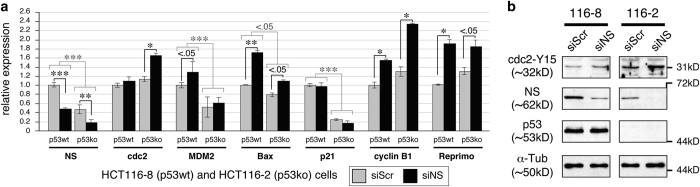
Molecular responses of p53wt and p53ko cancer cells to NS depletion. HCT116-8 (p53wt) and HCT116-2 (p53ko) cells were treated with siScr or siNS for 15 h and collected for analysis after 2 days. (**a**) The mRNA levels of p53-downstream genes were measured by qRT-PCR assays. (**b**) The amount of phosphorylated cdc2 (Y15), NS, and p53 in control (siScr) and NS-knockdown (siNS) HCT116-8 and HCT116-2 cells were measured by western blots. See Figure 1 for description regarding error bars and asterisks.

To determine whether p53 modulates the response of cancer cells to NSkd-induced G2/M arrest in the same way as it does in MEF cells, we analyzed the molecular responses of HCT116 cells to NSkd by qRT-PCR. The results showed that the baseline level of NS is two times higher in HCT116-8 cells than in HCT116-2 cells but the relative NSkd efficiencies are comparable in both cell lines (~60%). Similar to MEF cells, loss of NS upregulates the transcript levels of MDM2 in p53wt cells, cdc2 in p53ko cells, and Bax in both p53wt and p53ko cells, and does not change the p21 level in either p53wt or p53ko HCT116 cells (Figure 6a). Unlike MEF cells, NSkd increases cyclin B1 and reprimo expression in both p53wt and p53ko HCT116 cells. Western blots showed that NS depletion increases the amount of phosphorylated cdc2 (Y15) more in HCT116-2 cells than in HCT116-8 cells (Figure 6b). These data indicate that HCT-116 cells display a qualitative increase in phosphorylated cdc2, cyclin B1, and Bax in both p53wt and p53ko cells. Quantitatively, p53wt cells show more increase in reprimo and cyclin B1 and less increase in phosphorylated cdc2 compared with p53ko cells. Finally, normal and cancer cells show shared as well as unique molecular responses to NSkd-induced G2 arrest in contingency of their p53 status.

## Discussion

How NS intersects with the p53 pathway has always been a perplexing and highly debated issue in quest of the NS mechanism of action. This study illuminates this question by showing how p53 configures the response of cells to damages caused by the loss of NS. In our opinion, one major challenge in synthesizing a coherent model by integrating findings obtained from different cell models is to resolve their genetic heterogeneity. Such an issue exists not only between different cancer cell types but also between cancer and normal cells. To overcome this hurdle, we used two isogenic cell models with genetically perturbed NS and/or p53: one representing normal cells (e.g., MEF) and the other representing cancer cells (HCT116). This design permits the isogenic pairs to differ only in their NS and/or p53 status genetically.

### Semipermissive NS deficiency causes G2/M arrest regardless of p53

**Figure 7:**
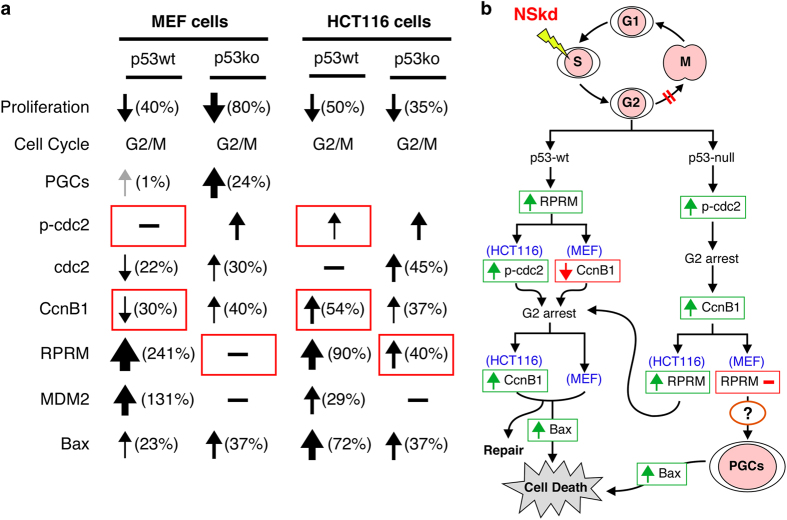
Diagrams of the p53-regulated response of NS-depleted cells to G2/M arrest. (**a**) Molecular and cellular responses of MEF and HCT116 cells to NS deficiency in the presence or absence of p53. Arrows and percentages in parenthesis denote the amount of increase (up arrow) or decrease (down arrow) induced by NS loss (NSkd). Red boxes mark the changes that are different in normal *versus* cancer cells. (**b**) A schematic diagram showing how NSkd affects cell cycling and how NS-deficient cells may respond to the resulting G2/M arrest under the p53wt or p53ko condition. Ccn B1, cyclin B1; PGCs, polypoid giant cells; RPRM, reprimo.

In both MEF cells and HCT116 cells, loss of NS triggers G2/M arrest and perturbs cell proliferation regardless of their p53 status. Because of its functional proximity to cell proliferation, NS was sometimes referred to as a cell cycle protein. We found that the level of NS protein remains constant throughout the cell cycle under the normal growth condition (Supplementary Figure S2), indicating that, at the level of protein expression, NS is neither regulating nor regulated directly by cell cycle progression. Depending on the extent of knockdown, NS-depleted cells may display the phenotypic profile of early S or G2/M arrest under the nonpermissive or semipermissive condition, respectively.^25^ The inNS^cko^ MEF cell model resembles the haploinsufficient MEF cells ^7^ in showing a semipermissive G2/M arrest phenotype. NSkd-triggered cell cycle arrest occurs before entry into mitotic prophase. Molecular analyses provide further insight into how p53 may shape the response of NS-deficient cells to G2/M arrest (Figure 7a). First, mitotic entry is propelled by cdc2 dephosphorylation and cyclin B1 upregulation. Both p53ko MEF and HCT116 cells show a significant increase in cyclin B1 and phosphorylated cdc2 following NS depletion, suggesting that failure to dephosphorylate cdc2 may be a driver and increased cyclin B1 may be a reactor in the event of NSkd-triggered G2/M arrest. With wild-type p53, the responses of cyclin B1 and phosphorylated cdc2 to NS loss are distinctive between normal and cancer cells, with HCT116 cells showing increases in both phosphorylated cdc2 and cyclin B1 and MEF cells showing no change in phosphorylated cdc2 and a decrease in cyclin B1. Some commonly known p53-downstream G2/M regulators (e.g., 14-3-3-*σ*, GADD45, and cyclin G1) appear not to participate in this process. Instead, the gene that is regulated by p53 and consistently activated by NS depletion in p53wt MEF and HCT116 cells is reprimo. Reprimo has been shown to be upregulated by X-ray irradiation in p53wt MEF cells and suitably involved in G2/M arrest.^28^ We therefore propose that NS deficiency arrests cells in the G2/M phase via a p53-dependent upregulation of reprimo or a p53-independent mechanism that blocks the dephosphorylation of cdc2.

### p53 reduces the frequency of aberrant mitosis in G2-arrested NS-depleted MEF cells

A combined loss of NS and p53 has a more detrimental effect on cell proliferation in normal (MEF) cells than loss of NS alone. This compounding effect of p53 deletion in NS-depleted cells cannot be accounted for by the rather minor increase of cellular senescence or cell cycle perturbation. Instead, this proliferative crisis is most quantitatively compatible with a dramatic increase of PGCs, which suggests that p53 loss may subject the G2-arrested, NS-depleted cells to endoreplication and mitotic catastrophe. We reason that the molecules suppressing this event should be active in p53wt MEF cells and disabled in p53ko MEF cells. They should also be active in HCT116-8 and HCT116-2 cells, because the compounding effect of p53 deletion is not seen in HCT116 cells. Among the genes examined, only reprimo fits this profile, where its expression is upregulated by NSkd in p53wt MEF cells, HCT116-8 cells, and HCT116-2 cells, but remains unchanged in p53ko MEF cells. These results suggest a potential hypothesis that upregulation of reprimo in response to p53 activation following NS depletion may prevent cells from undergoing endoreplication and becoming PGCs, thereby reducing the risk of mitotic catastrophe.

### p53-independent regulation of reprimo and Bax

In the context of NS perturbation, the expression of MDM2, p21, and cyclin G1 shows absolute p53 dependence, in the sense that they display a higher expression levels in p53wt than in p53ko cells. They either do not respond to NSkd specifically (e.g., p21 and cyclin G1) or respond to NSkd in a p53-dependent manner (e.g., MDM2). Conversely, cyclin A2, p27^kip^, and cdc25C exhibit a p53-inhibited, NS-independent pattern of expression. NSkd increases Bax expression in both normal and cancer cells regardless of their p53 status. Notably, the expression of reprimo in HCT116 cells can also be increased by NSkd in HCT116-2 (p53ko) cells. Indeed, it has been reported that the expression of reprimo can be regulated by promoter methylation as well as other p53-independent mechanisms in some cancer cells.^29–31^

### Differential responses of normal and cancer cells to NS/p53 loss

Notably, MEF and HCT116 cells exhibit some differential responses to NS/p53 perturbation. For example, the NSkd-triggered reprimo response is p53-dependent in MEF cells and p53-independent in HCT116 cells. The cyclin B1 response to NSkd is decreased in p53wt MEF cells and increased in HCT116-8 cells. Another difference between MEF and HCT116 cells relates to their NS expression. In MEF cells, p53 deletion does not affect the basal expression level of NS but appears to sensitize them to TAM-induced NS depletion. Contrarily, loss of p53 reduces the baseline level of NS without affecting their respective sensitivities to NSkd. The exact mechanisms that drive the differential responses of cancer *versus* normal cells to NS/p53 perturbation remain to be determined on a case-by-case basis, but may relate to other cancer-associated mutations, such as p16 inactivation in HCT116 cells. Furthermore, even though the genetic variables of the isogenic cell pairs used in this study are limited to NS and p53 only, they may develop different adaptive responses that alter their expression of genes beyond NS and p53. Finally, there are likely multiple steps in between the NS/p53 perturbation and the readout events (e.g., G2/M arrest, cell death, and PGCs). Nevertheless, given the key positions where NS and p53 sit in cell proliferation and checkpoint monitoring, respectively, they may serve as a valuable predictor of the proliferative fate of normal and cancer cells. Finally, the different responses of normal and cancer cells to the perturbation of NS and/or p53 may potentially be used to design cancer-specific therapies.

## Conclusion

Our data show that even though p53 loss cannot reverse the damage caused by NS loss or the ultimate fate of NS-deficient cells, it will orchestrate how they respond to the G2/M arrest (Figure 7b). The presence of p53 provides a mechanism for normal G2-arrested cells to reduce the chance of mitotic catastrophe. On the other hand, cancer cells appear to manage the NS loss condition more independently of their p53 status. The differential response of normal and cancer cells to NS deficiency may provide a rationale for developing tumor-selective therapies by targeting the NS activity.

## Materials and methods

### Cell culture, RNAi knockdown, and synchronization

Genetically modified mouse models were created and described in a previous report.^9^ Animals were housed by the Program for Animal Resources at the TAMHSC-Houston campus and handled in accordance with the principles of the Guide for the Care and Use of Laboratory Animals. All procedures were approved by the Institutional Animal Care and Use Committee. MEF cells were prepared from NS^flx/flx^-p53wt, NS^flx/flx^-p53ko, inNS^cko^-p53wt, and inNS^cko^-p53ko embryos at embryonic day 13.5 using the method shown previously.^7^ The flow cytometry (Figures 2a and 3b), mitosis (Figure 2b), and qRT-PCR (Figure 4) studies were performed by using the second passage (P2) MEF cells. The SA-β-Gal staining (data not shown) and giant cell studies (Figure 3a) were conducted in P3 MEF cells. HCT116-8 and HCT116-2 cells were obtained from Dr. Bert Vogelstein and cultured in McCoy’s 5 A medium plus 10% FBS. HeLa cell lines were maintained in DMEM plus 10% FBS. For NSkd, HCT116 cells were replated for 24 h and then treated with siRNA duplexes (siScr or siNS) at 100 nM in Oligofectamine complex (Invitrogen, Carlsbad, CA, USA) for 15 h. The RNA sequences targeted by the siRNA duplexes are 5′-UGA CGA UCA GAA UGC GAC U-3′ (siScr) and 5′-GAA CUA AAA CAG CAG CAG A-3′ (siNS). Early S-phase synchronization was achieved by incubating HeLa cells with 2 mM thymidine for 20 h. Mitotic arrest was done by incubating cells with 0.5 *μ*M nocodazole for 20 h.

### Flow cytometry

At the time of the analysis, cells were washed, trypsinized, and fixed in 72% (vol/vol) ice-cold ethanol overnight. Cells were first processed for propidium iodide staining (50 μg/ml, Sigma, St. Louis, MO, USA) in the presence of 20 μg/ml DNase-free RNase A. Flow cytometry analyses were conducted using a COULTER EPICS XL flow cytometer and the XL System II software in the Flow Cytometry Core Facility at the Texas Children’s Hospital. Cell cycle profiles were compiled from 2×10^4^ gated events and analyzed using the Multi Cycle AV software. All data represent the average of⩾4 independent repeats.

### Western blot

Western blot analyses were performed as described previously and repeated twice.^14,32^ Primary antibodies used in this study include rabbit polyclonal antibodies to human NS (Ab138, Tsai Laboratory, Houston, TX, USA), p-cdc2 (Y15, Cell Signaling, Danvers, MA, USA), MDM2 (SMP-14, Santa Cruz, Santa Cruz, CA, USA), p53 (DO-1, Santa Cruz), p-histone H3 (S10, Upstate, Lake Placid, NY, USA), and α-tubulin (Sigma). Secondary antibodies were conjugated to peroxidase (Jackson ImmunoResearch, West Grove, PA, USA).

### Anti-pH3 staining

To measure the percentages of cells in different mitotic phases, cells were fixed in 4% formaldehyde for 15 min, permeablized by 0.3% Triton X-100, and incubated with anti-p-histone H3 antibody overnight, followed by incubation with FITC-conjugated secondary antibodies for 2 h. Abnormal nuclear morphologies were analyzed by DAPI staining. Fluorescent images were acquired on a Zeiss LSM510 confocal microscope (Göttingen, Germany) using the ×40 or ×63 plan-apochromat objectives. Images were scanned with a 512×512 frame size and ×1 zoom. Detector gain and amplifier offset were adjusted to ensure that all signals were displayed within the linear range. Final data represent the average of three independently performed experiments.

### Quantitative RT-PCR analyses

Total RNAs (5μg) were reversed transcribed into first-strand cDNAs using random hexamers and M-MLV reverse transcriptase. For qPCR, the ΔC(t) values between the target message and the reference message (Rplp0) were determined by the MyiQ single-color real-time PCR detection system and supermix SYBR green reagent. The ΔΔC(t) values were measured from three biological replicates and two technical repeats (*n*=6) to compare the relative expression levels of target sequences between different groups. All final results were confirmed by comparing to a second reference message, HMG-14. Primer sequences are provided in Supplementary Data.

### Clonogenic survival assay

HCT116 cells were treated with siRNAs for 24 h, replated at low density (55 cells/cm^2^) in six-well plates at a density of 300 cells per well, and grown in normal growth medium for 12 days to allow colony formation. Formed colonies were then visualized by fixing with 3.7% formaldehyde for 15 min at room temperature and staining with 0.25% crystal violet for 30 min. Colonies larger than 1mm in diameter were counted. Final data represent the average of three independently performed experiments and are analyzed by Repeated Measures ANOVA.

## Abbreviations

MEF: mouse embryonic fibroblast
NS: nucleostemin
NSkd: NS-knockdown
PGCs: polyploid giant cells
PI: propidium iodide
pH3: phospho-histone H3
p53wt: p53-wildtype
p53ko: p53-null.
