# Supplementary material for: p53 Configures the G2/M arrest response of nucleostemin-deficient cells
Source: Cell Death Discov. 2015 Nov 23;1:15060–. doi: 10.1038/cddiscovery.2015.60 (PMC4729372; doi:10.1038/cddiscovery.2015.60)
Supplement: Supplementary Material [file cddiscovery201560-s1.doc]

**SUPPLEMENTAL DATA**

**Materials and Methods**

**Primer sequences for qRT-PCR assay**

1. Mouse NS (56C), 5’-GTC TGA TCT AGT ACC AAA GG-3’; 5’-GGG AAA CCA ATC ACT CCA AC-3’

2. Mouse cyclin E1 (56C), 5’-GAG ATG AGC ACT TTC TGC AGC-3’; 5’-AGT CCT GTG CCA AGT AGA ACG-3’

3. Mouse cyclin A2 (56C), 5’-ATG CAG CTG TCT CTT TAC CCG-3’; 5’-CCT CCA TTT CCC TAA GGT ACG-3’

4. Mouse p27 (56C), 5’-CCA GAC GTA AAC AGC TCC GAA-3’; 5’-GGC AGA TGG TTT AAG AGT GCC-3’

5. Mouse p21 (59C), 5’-TGT CTT GCA CTC TGG TGT CTG-3’; 5’-GAG TGA TAG AAA TCT GTC AGG C-3’

6. Mouse cdc2 (56C), 5’-CCT GGG CAG TTC ATG GAT TCT-3’; 5’-AGG CCG AAA TCA GCC AGT TTG-3’

7. Mouse cyclin B1 (56C), 5’-TGA ACC AGA GGT GGA ACT TGC-3’; 5’-AGA TGT TTC CAT CGG GCT TGG-3’ (?)

8. Mouse cdc25C (59C), 5’-AAC GCC ATT CAG ATG GAG GAG-3’; 5’-TTT CCA GAC AGC AAA GCA GCC-3’

9. Mouse 14-3-3-σ (59C), 5’-AAC AGG CCG AAC GGT ATG AAG-3’; 5’-CCA CGT TCT TGT AAG CTA CGG-3’

10. Mouse GADD45 (59C), 5’-CAG AAG ACC GAA AGG ATG GAC-3’; 5’-CGT TAT CGG GGT CTA CGT TGA-3’

11. Mouse reprimo (56C): 5’-CAG CCT GTA CAT CAT GCG TGT-3’, 5’-GTT GAT CAT GCC TTC GGA CTT G-3’

12. Mouse cyclin G1 (56C): 5’-GCC CAT GAT AAT GGC CTC AGA-3’, 5’-CTC AGT CCA ACA CAC CCA AGA-3’

13. Mouse MDM2 (56C), 5’-GAG TTT CTC TGT GAA GGA GCA-3’; 5’-GAA GGT TTC TCT TCT GGT GGC-3’

14. Mouse Bax (56C), 5’-TGG AGC TGC AGA GGA TGA TTG-3’; 5’-GCA AAG TAG AAG AGG GCA ACC-3’

15. Mouse Apaf-1 (56C), 5’-GCA AAC ACC CAA GGT CTC TGT-3’; 5’-GGA CAA CAT GCT TAG GAC CCA-3’

16. Mouse p63 (59C), 5’-ATG CTC AGT ACA GCC CAT CGA-3’; 5’-TCT GAA TCT GCT GGT CCA TGC-3’

17. Mouse Rplp0 (55-64C), 5’-CTG AAG TGC TCG ACA TCA CA-3’; 5’-AGT CTC CAC AGA CAA TGC CA-3’

18. Human NS (56C), 5’-GAA CAA AGC CAA GTC GGG-3’; 5’-GTC CAC TCT GGA CAA TGG-3’

19. Human p21 (56C), 5’-ACC ATG TGG ACC TGT CAC TGT-3’; 5’-GAG TGG TAG AAA TCT GTC ATG C-3’

20. Human cdc2 (56C), 5’-CTA GAA GAG TTC TTC ACA GAG AC-3’; 5’-GGA GTT GAG TAA CGA GCT GAC-3’

21. Human cyclin B1 (59C), 5’-CTG AGA CAA CTT GAG GAA GAG C-3’; 5’-ACA TGG TCT CCT GCA ACA ACC-3’

22. Human reprimo (56C): 5’-GAC GAG CGT AGC CTG TAC ATA-3’, 5’-TTG ATC ATG CCC TCG GAC TTG-3’

23. Human MDM2 (56C), 5’-TCT CTG TGA AAG AGC ACA GGA A-3’; 5’-CTG AAG CTC TTG TAC AAG GTC C-3’

24. Human Bax (56C), 5’-TGC TTC AGG GTT TCA TCC AGG-3’; 5’-CAA TCA TCC TCT GCA GCT CCA-3’

25. Human Rplp0 (54-61C), 5’-ACC CTG AAG TGC TTG ATA TCA C-3’; 5’-AAT CCG TCT CCA CAG ACA AGG-3’

**Figure Legends**

Figure S1. MEF cell models with NS and/or p53 perturbation.

(**a**) Anti-NS and α-tubulin (α-Tub) western blots of NS^flx/flx^ or inNS^cko^ MEF (p53wt) cells at P2 and P3. Cells were treated with DMSO (control) or TAM. (**b**) Ratios of proliferative potential between p53ko and p53wt cells under the NS-wildtype (NSwt, black line) and NS-knockdown (NSkd, grey line) conditions from P1 to P4.

Figure S2. NS protein level is not changed during cell cycle progression.

HeLa cells were synchronized in early S phase and collected after release for 0 to 20 hours with a 2-hour interval. Western blots of NS, phospho-histone H3 (pH3), and α-Tub were shown as indicated. Unsynchronized (I) and mitotic shake-off of nocodazole-treated (M) cells were included.
