# Supplementary figures and images for: p53 Configures the G2/M arrest response of nucleostemin-deficient cells
Source: Cell Death Discov. 2015 Nov 23;1:15060–. doi: 10.1038/cddiscovery.2015.60 (PMC4729372; doi:10.1038/cddiscovery.2015.60)

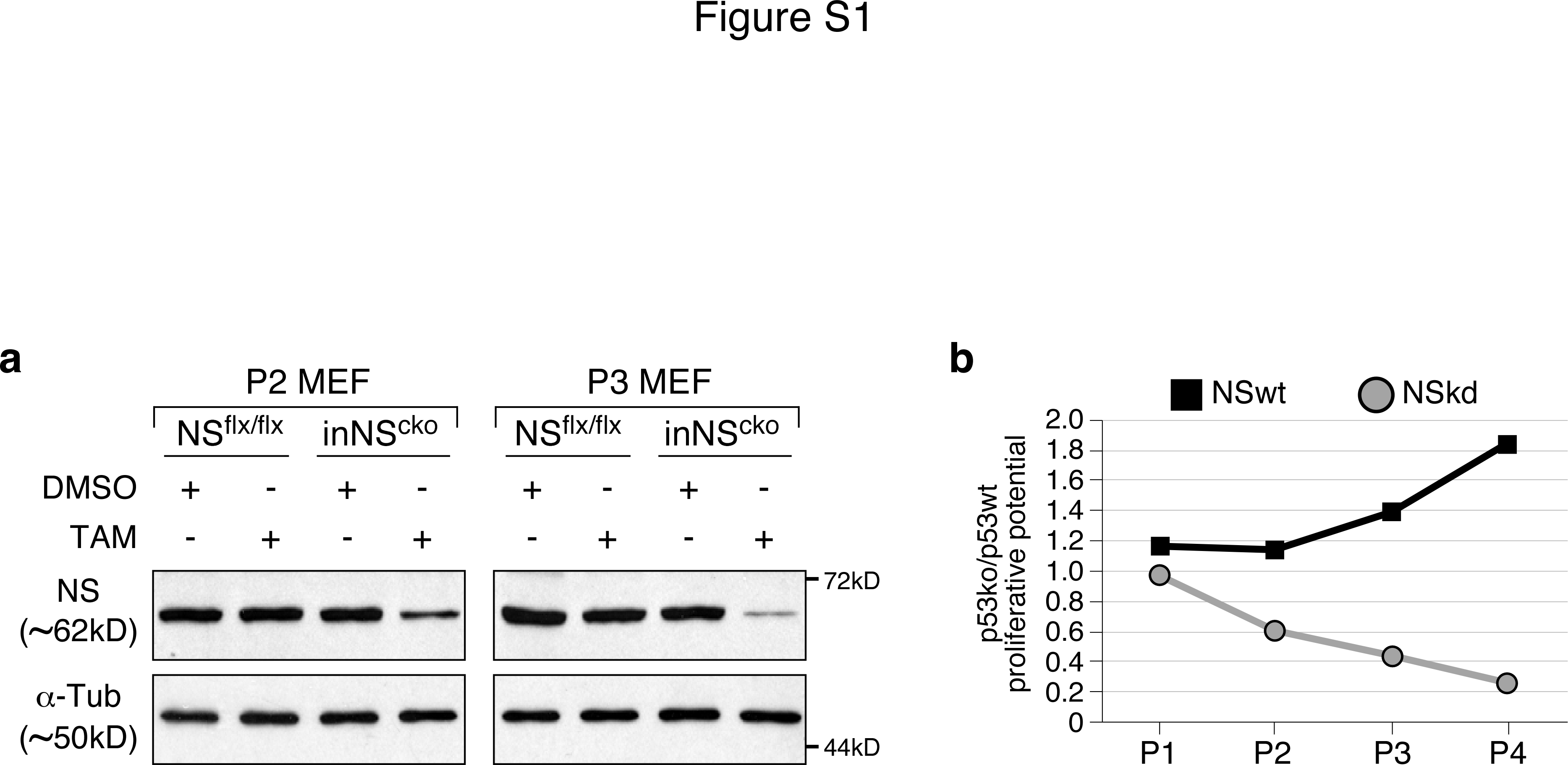

Supplement: Supplementary Figure S1 [file cddiscovery201560-s2.tiff]

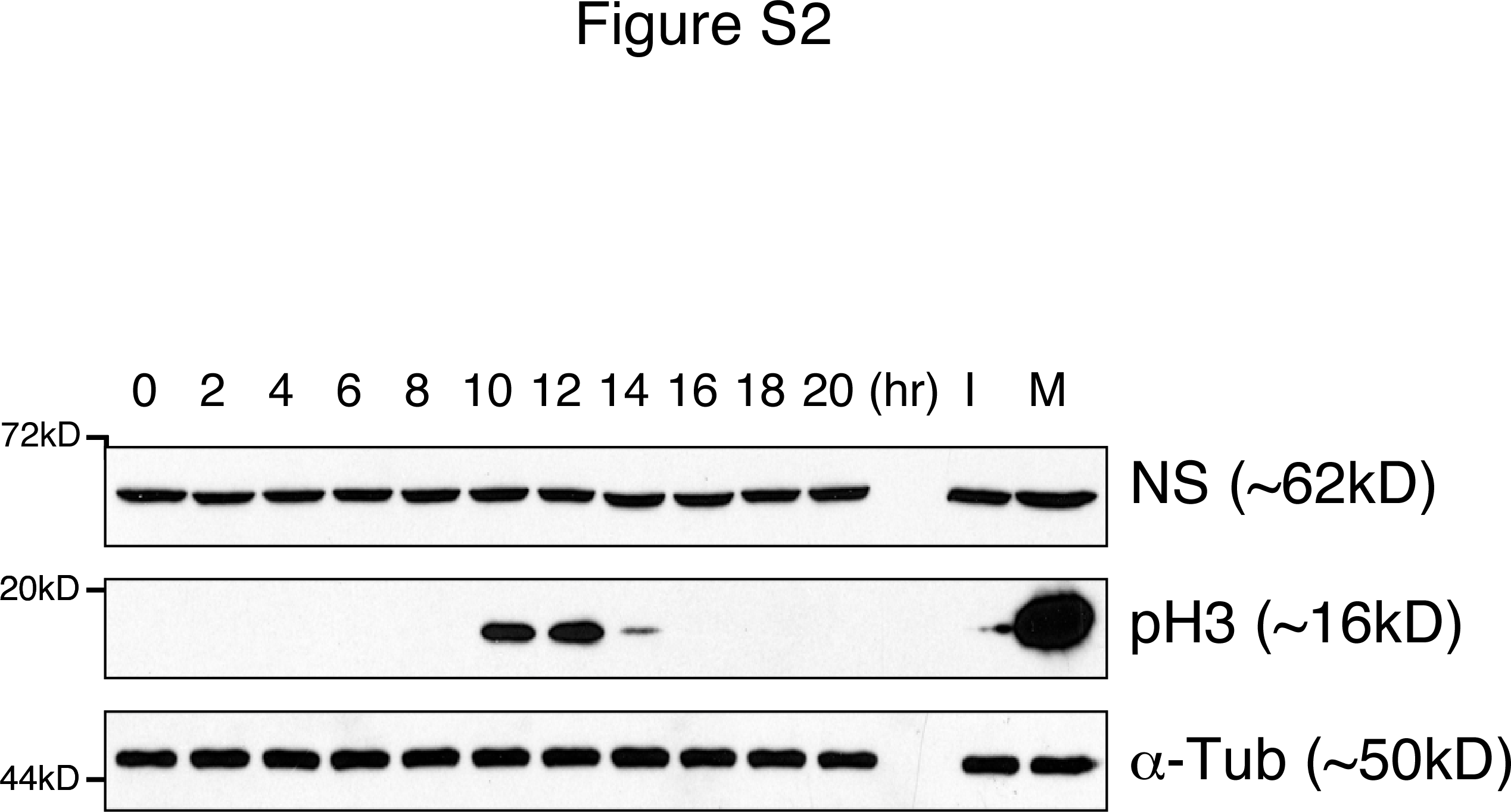

Supplement: Supplementary Figure S2 [file cddiscovery201560-s3.tiff]
